# Deep placement of controlled-release urea effectively enhanced nitrogen use efficiency and fresh ear yield of sweet corn in fluvo-aquic soil

**DOI:** 10.1038/s41598-019-56912-y

**Published:** 2019-12-30

**Authors:** Wei Liu, Yousheng Xiong, Xiangyu Xu, Fangsen Xu, Saddam Hussain, Hanfeng Xiong, Jiafu Yuan

**Affiliations:** 1 Key Laboratory of Fertilization from Agricultural Wastes, Ministry of Agriculture and Rural Affairs/Institute of Plant Protection and Soil Fertilizer, Hubei Academy of Agricultural Science, Wuhan, 430064 China; 2 College of Resources and Environment, Huazhong Agricultural University, Wuhan, 430070 China; 3 Department of Agronomy, University of Agriculture, Faisalabad, 38040 Pakistan; 4 Ezhou Polytechnic, Ezhou, Hubei 436000 China

**Keywords:** Plant sciences, Environmental sciences

## Abstract

Application of controlled-release urea (CRU) improves crop yield and nitrogen use efficiency (NUE) compared with conventional urea. However, the effectiveness of CRU differs with fertilization placement. A two site-year field experiment was carried out in fluvo-aquic soil in central China to study the effects of two N sources (CRU and urea) and three fertilization placements (band application between two corn rows at 0, 5, and 15 cm soil depths) on fresh ear yield and NUE of sweet corn. The soil inorganic N (NO_3_^−^-N and NH_4_^+^-N) concentrations at the soil layers of 0–20 cm and 20–40 cm, root morphology characteristics and leaf physiological functions were also measured during the sweet corn growth period. Results showed that the deep placement of CRU at 15 cm soil depth significantly increased the sweet corn fresh ear yield, total N uptake, and NUE by 6.3%–13.4%, 27.9%–39.5%, and 82.9%–140.1%, respectively compared with CRU application at 0 cm depth. Deep placement of CRU at 15 cm also increased the root morphology traits, gas exchange attributes, and soil NO_3_^−^-N and NH_4_^+^-N concentrations in 0–20 cm and 20–40 cm layer, especially during later crop growth stages. However, the different N placements exerted non-significant effects on NUE and fresh ear yield when urea was applied as the N source. In crux, deep CRU placement instead of urea at 15 cm depth can effectively improve fresh ear yield and NUE of sweet corn in fluvo-aquic soil because of higher root growth, better leaf physiological functions and increased availability of soil NO_3_^−^-N and NH_4_^+^-N.

## Introduction

Sweet corn (*Zea mays* L.) cultivation is receiving great attention in China and across the globe because of its high sugar content and unique combination of flavor, texture, and aroma compared with other types of corn^1,2^. Sweet corn is also an important source of fiber, minerals, and vitamins^3,4^. The planting areas of sweet corn have rapidly increased in China with the progress of urban agriculture and adjustment of agricultural structure^5^. Nitrogen (N) is an essential element for plant growth and development^6,7^. Excessive amounts of N fertilizer have been used in China to achieve high yields of field crops, including sweet corn^8^. Nevertheless, excessive N application not only decreases N use efficiency (NUE) but also increases N losses from field soils to the environment through various pathways, thereby deteriorating air, water, and soil qualities^9,10^. Therefore, improved fertilizer management practices that simultaneously maximize crop yield, minimize environmental impacts, and maintain soil productivity should be explored^11,12^.

Effective fertilizer placement needs excellent timing according to the demand of the crops and environmental conditions with low risk of nutrient losses. Split fertilizer placement during important growth stages of the crop enhances the uptake of nutrient and final yield^13,14^. However, applying the appropriate rate and quantity manually is difficult for farmers because it requires time and additional labor costs. The rapid economic development in China has moved rural laborers to developed coastal areas^15^, resulting in the lack of human labor and its high cost. Therefore, a simple and convenient N fertilizer management is needed for sweet corn production.

The development of a controlled-release N fertilizer technique has potential in addressing the aforementioned issues^16^. Controlled-release urea (CRU) is introduced to famers as a new type of N fertilizer for agricultural productions in China, the USA, and even worldwide^17^. CRU is widely used as an effective mitigation alternative in improving crop productivity and reducing labor inputs^18^. CRU provides a gradual N supply as per crop requirement for prolonged period^7,19^. The N release of CRU coordinates with the N requirement pattern of various crops^7,20–22^, thereby reducing N loss and enhancing soil inorganic N supply, especially at later growth periods^23,24^. The differences in the environmental conditions can also affect the characteristics of N release and its coordination with crop demand^25^. For example, a previous study on ^15^N-labeled reported higher maize yield and NUE with CRU than urea^26^, but some other researchers have reported contrasting results^27,28^. The different responses of crop yields to CRU suggest the need for further research in order called for additional investigation is required to determine the most effective application method and placement depth in different environmental conditions.

The deep placement of fertilizers, especially urea, efficiently abates N loss, particularly ammonia volatilization, and increases crop yield and NUE^29–31^. Similar to conventional urea, deep CRU placement effectively increases the productivity of various crops, such as soybean^32^, summer maize^33^, and rice^34^. However, the effects of CRU fertilization with different placements on fresh ear yield and NUE in a sweet corn field remain poorly understood. In the present study, a two-year field experiment was performed to investigate the effect of two N sources, namely, CRU and urea, and three fertilization placement depths on crop N uptake, NUE, and fresh ear yield in sweet corn in fluvo-aquic soil in central China. The specific objectives of this study included (1) evaluating the effects of CRU application with varying placement depths on fresh ear yield and NUE compared with conventional urea application and (2) comparing the dry matter accumulation and N uptake of the aboveground parts, root morphology characteristics, and soil inorganic N (NO_3_^−^-N and NH_4_^+^-N) concentrations during the main sweet corn growth stages under different N treatments.

## Results

### Cumulative release rate of N from CRU in 25 °C water

**Figure 1:**
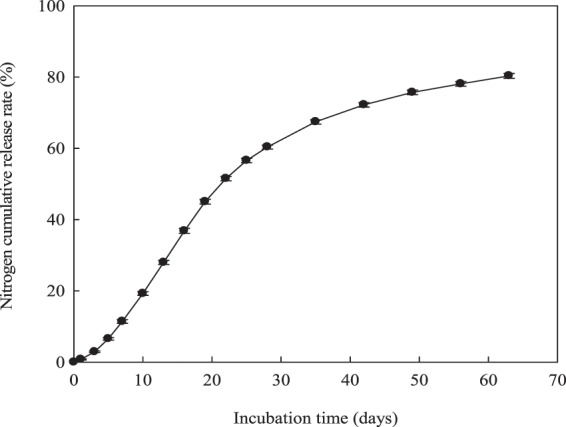
Nitrogen-accumulated release rate dynamics of CRU in water at 25 °C.

The N cumulative release rate of CRU followed an “S” shape curve under laboratory conditions (25 °C still water), as shown in Fig. 1. The N release of CRU was slow during the first 24 h, and the N cumulative release rates reached only at 0.7%. This slow release stage indicated that the membrane structure of CRU was relatively complete and that the controlled-release fertilizer had good performance. An accelerated release stage (1–22 days) followed, finishing with a reduced N release stage after 22 days. The N cumulative release rates for CRU reached 80.3% in 63 days.

### Fresh ear yield and NUE

Relative to N0, urea application significantly (*P* < 0.05) increased fresh ear yield by 13.1% (11.3%–15.8%) in 2016 and 12.9% (11.8%–14.1%) in 2017. The CRU application also significantly (*P* < 0.01) increased fresh ear yield by 13.6% (7.8%–22.2%) in 2016 and 11.9% (8.2%–15.0%) in 2017 (Table 1). The highest fresh ear yield was achieved at D15 placement for both N sources. When CRU was applied, the fresh ear yield showed an increasing trend with the increase in fertilization depth from 0 cm (D0) to 15 cm (D15). The fresh ear yield was significantly (*P* < 0.05) increased by 13.4% and 6.3% in the CRU application at D15 placement during 2016 and 2017, respectively compared with D0 placement. However, the differences were non-significant (*P* > 0.05) in both years among the D0, D5, and D15 placements when urea was applied as the N source.

**Table 1:** Effects of nitrogen (N) source and placement on fresh ear yield and N use efficiency (NUE) of sweet corn in 2016 and 2017.

Placement	N source	Fresh ear yield (t ha^−1^)			NUE (%)		
		2016	2017	Mean	2016	2017	Mean
	N0	12.19 c	15.30 c	13.74 d			
D0	Urea	13.69 b	17.11 ab	15.40 bc	11.8 c	19.4 c	15.6 c
	CRU	13.14 b	16.56 b	14.85 c	18.2 bc	21.6 c	19.9 c
D5	Urea	13.57 b	17.25 ab	15.41 bc	13.9 bc	21.3 c	17.6 c
	CRU	13.48 b	17.19 ab	15.34 bc	23.4 b	32.1 b	27.8 b
D15	Urea	14.12 ab	17.45 a	15.79 ab	20.0 bc	21.5 c	20.8 c
	CRU	14.90 a	17.60 a	16.25 a	43.7 a	39.5 a	41.6 a
**ANOVA**							
N source		Ns	Ns	Ns	**	**	**
Placement		*	*	**	**	**	**
N source × placement		Ns	Ns	Ns	Ns	**	**

Data are presented as the means of three replications. Within a column for each season, values followed by different letters indicate significant difference according to Duncan’s test at *P* = 0.05. **P < 0.01; *P < 0.05; Ns, *P* > 0.05.

N source and placement exerted significant effects on NUE in both years (*P* < 0.01). The interaction between the N sources and placement was also significant (*P* < 0.01) in 2017 (Table 1). With both N sources, NUE presented an increasing trend as fertilization depth was increased from D0 to D15. Across both years, CRU application at D5 and D15 placements increased the NUE significantly (*P* < 0.01) compared with urea application at the same placement depths. However, both urea and CRU were statistically similar (*P* > 0.05) to each other at D0 (Table 1).

### Dry matter and N accumulation

Both N source and placement had non-significant effect (*P* > 0.05) on the aboveground dry matter accumulation at the V6 stage in 2016 and 2017 (Table 2). However, the individual effect of the N source for the aboveground dry matter accumulation from V6 to R3 stages in 2016 (*P* < 0.05) and from VT to R3 stages in 2017 was significant (*P* < 0.01). The effect of N placement was significant (*P* < 0.01) for aboveground dry matter accumulation from VT to R3 stages in 2016 and from V6 to R3 stages in 2017. The interactive effect of N source and placement was also significant (*P* < 0.05) for dry matter accumulation from VT to R3 stages in both years. Compared with N0 treatment, urea application significantly (*P* < 0.01) increased the dry matter accumulation from V6 to R3 stages by 9.0%–21.5% in 2016 and 23.6%–27.8% in 2017. CRU application also significantly (*P* < 0.01) increased dry matter accumulation from V6 to R3 stages by 22.0%–64.1% in 2016 and 17.9%–36.3% in 2017. Compared with urea application, CRU application increased dry matter accumulation significantly (*P* < 0.05) from V6 to R3 stages by 9.1%, 16.1%, and 35.1% at D0, D5, and D15 placements in 2016, respectively. Compared with D0 placement, urea application at D15 placement increased dry matter accumulation after V6 stage by 8.6% and 3.4% in 2016 and 2017 (*P* > 0.05), respectively. However, the corresponding increase in dry matter accumulation after V6 stage was 34.5% and 15.6% by CRU application in 2016 and 2017, respectively (*P* < 0.05). CRU application at D15 placement resulted in the highest dry matter accumulation rate from V6 to R3 stages in 2016 and from VT to R3 stages in 2017.

**Table 2:** Differences in aboveground dry matter weight and accumulation rate during main sweet corn growth stages under different N fertilization treatments in 2016 and 2017.

Year	Placement	N source	Aboveground dry matter (kg ha^−1^)			Dry matter accumulation rate (kg ha^−1^ day^−1^)		
			V6	VT	R3	Before V6	V6-VT	VT-R3
2016		N0	180.3 a	2058.1 c	8031.8 c	7.2 a	75.1 c	238.9 b
	D0	Urea	234.7 a	2528.9 abc	9013.1 bc	9.4 a	91.8 abc	259.4 b
		CRU	226.4 a	2943.1 a	9803.1 b	9.1 a	108.7 a	274.4 b
	D5	Urea	215.8 a	2221.1 bc	8771.3 bc	8.6 a	80.2 bc	262.0 b
		CRU	250.5 a	2749.1 ab	10183.3 b	10.0 a	99.9 abc	297.4 b
	D15	Urea	221.8 a	2765.7 ab	9759.3 b	8.9 a	101.8 ab	279.7 b
		CRU	222.5 a	3031.9 a	13102.9 a	8.9 a	112.4 a	402.8 a
	ANOVA							
	N source		Ns	*	**	Ns	*	**
	Placement		Ns	Ns	**	Ns	Ns	**
	N source × placement		Ns	Ns	Ns	Ns	Ns	*
2017		N0	162.4 c	3337.4 d	5814.1 d	6.2 c	132.3 d	85.4 d
	D0	Urea	256.9 ab	3643.5 cd	7244.1 bc	9.9 ab	141.1 cd	124.2 ab
		CRU	273.0 ab	3733.0 cd	6935.4 c	10.5 ab	144.2 cd	110.4 abcd
	D5	Urea	291.0 a	4426.0 ab	7382.4 bc	11.2 a	172.3 ab	101.9 bcd
		CRU	257.6 ab	4071.0 bc	7489.2 b	9.9 ab	158.9 bc	117.9 abc
	D15	Urea	216.7 b	4706.8 a	7438.2 b	8.3 b	187.1 a	94.2 cd
		CRU	259.4 ab	4040.9 bc	7963.0 a	10.0 ab	157.6 bcd	135.2 a
	ANOVA							
	N source		Ns	Ns	**	Ns	**	Ns
	Placement		Ns	**	**	Ns	**	Ns
	N source × placement		Ns	Ns	**	Ns	Ns	*

Data are presented as the means of the three replicates. Within a column for each season, values followed by different letters indicate significant difference according to Duncan’s test at *P* = 0.05. V6: sixth leaf stage; VT: tasseling stage; R3: harvest (milk maturity) stage. **P < 0.01; *P < 0.05; Ns, *P* > 0.05.

Similarly, N source had a significant effect on the aboveground N accumulation from V6 to R3 stages in 2016 (*P* < 0.05) and from VT to R3 stages in 2017 (*P* < 0.01). Placement had a significant effect on the aboveground N accumulation from VT to R3 stages in 2016 (*P* < 0.01) and from V6 to R3 stages in 2017 (*P* < 0.05) (Table 3). The highest N accumulation at the VT stage in 2016 was observed in CRU application at D15 placement. Urea application at D15 placement recorded the highest N accumulation at the VT stage in 2017, but the difference was non-significant (*P* > 0.05) between the CRU application at D5 and D15 placements. Consistent to dry matter accumulation rate, CRU application at D15 placement also resulted in the highest N accumulation rate from V6 to R3 stages in 2016 and from VT to R3 stages in 2017.

**Table 3:** Effect of N source and placement on aboveground N accumulation amount and rate during main sweet corn growth stages in 2016 and 2017.

Year	Placement	N source	N accumulation amount (kg ha^−1^)			N accumulation amount rate (kg ha^−1^ day^−1^)		
			V6	VT	R3	Before V6	V6 to VT	VT to R3
2016		N0	5.4 a	21.8 d	83.1 d	0.22 a	0.66 d	2.45 d
	D0	Urea	8.0 a	38.0 b	104.4 c	0.32 a	1.20 ab	2.66 cd
		CRU	7.3 a	34.5 b	115.9 bc	0.29 a	1.09 bc	3.25 bcd
	D5	Urea	7.3 a	29.5 c	108.1 bc	0.29 a	0.89 cd	3.14 bcd
		CRU	8.0 a	35.6 b	125.2 b	0.32 a	1.11 bc	3.58 b
	D15	Urea	7.6 a	33.8 bc	119.0 bc	0.30 a	1.05 bc	3.41 bc
		CRU	7.8 a	43.3 a	161.7 a	0.31 a	1.42 a	4.74 a
	ANOVA							
	N source		Ns	*	**	Ns	Ns	**
	Placement		Ns	Ns	**	Ns	Ns	**
	N source × placement		Ns	Ns	Ns	Ns	Ns	Ns
2017		N0	5.6 d	58.9 d	77.0 d	0.22 d	2.22 d	0.62 e
	D0	Urea	11.1 ab	73.2 c	112.0 c	0.43 ab	2.59 cd	1.34 bc
		CRU	11.5 ab	76.0 bc	115.9 c	0.44 ab	2.69 bcd	1.37 bc
	D5	Urea	12.6 a	82.6 bc	115.3 c	0.48 a	2.92 bc	1.13 cd
		CRU	9.7 bc	87.4 ab	134.8 b	0.37 bc	3.24 ab	1.63 b
	D15	Urea	7.7 cd	95.1 a	115.8 c	0.30 cd	3.64 a	0.71 de
		CRU	10.4 ab	83.8 ab	148.2 a	0.40 ab	3.06 bc	2.22 a
	ANOVA							
	N source		Ns	Ns	**	Ns	Ns	**
	Placement		*	**	**	*	**	Ns
	N source × placement		*	Ns	**	*	Ns	**

Data are presented as the means of three replicates. Within a column for each season, values followed by different letters indicate significant difference according to Duncan’s test at *P* = 0.05. V6: sixth leaf stage; VT: tasseling stage; R3: harvest (milk maturity) stage. **P < 0.01; *P < 0.05; Ns, *P* > 0.05.

### Root morphological characteristics

Data on root morphology characteristics, including root length, surface area, and volume at the VT stage, are provided in Table 4. The N placement had a significant effect on root surface area and volume at the VT stage in 2016 (*P* < 0.05) and on root length, surface area, and volume at the VT stage in 2017 (*P* < 0.05). The individual effect of N source and the interaction between N source and placement had non-significant effect (*P* > 0.05) on the root morphology attributes in both years. The highest values for these root morphological parameters at the VT stage were observed in CRU application at D15 placement in both years.

**Table 4:** Root morphological characteristics of sweet corn at VT stage in 2016 and 2017.

Year	Placement	N source	Root length (cm plant^−1^)	Root surface area l (cm^2^)	Root volume (cm^3^ plant^−1^)
2016		N0	1715.7 b	915.1 b	23.8 c
	D0	Urea	1880.3 b	904.5 b	26.8 bc
		CRU	2239.9 ab	867.9 b	27.0 bc
	D5	Urea	2258.6 ab	963.0 b	27.4 bc
		CRU	2372.3 ab	945.7 b	30.5 bc
	D15	Urea	2725.2 a	1070.4 ab	37.1 ab
		CRU	2803.2 a	1286.1 a	47.3 a
	ANOVA				
	N source		Ns	Ns	Ns
	Placement		Ns	*	**
	N source × placement		Ns	Ns	Ns
2017		N0	2660.4 c	919.1 c	35.8 d
	D0	Urea	3755.4 bc	1261.8 bc	39.9 cd
		CRU	4213.8 ab	1337.6 ab	38.6 cd
	D5	Urea	5605.6 a	1604.5 ab	43.0 bcd
		CRU	4752.5 ab	1540.9 ab	44.4 bc
	D15	Urea	5330.7 a	1651.4 a	50.0 ab
		CRU	5664.3 a	1678.7 a	53.1 a
	ANOVA				
	N source		Ns	Ns	Ns
	Placement		*	*	**
	N source × placement		Ns	Ns	Ns

Data are presented as the means of three replicates. Within a column for each season, values followed by different letters indicate significant difference according to Duncan’s test at *P* = 0.05. VT, tasseling stage. **P < 0.01; *P < 0.05; Ns, *P* > 0.05.

### Leaf physiological functions

The leaf area index (LAI), net photosynthetic rate (Pn), stomatal conductance (Gs), transpiration rate (Tr), and SPAD value of sweet corn at the VT stage are presented in Table 5. N source had a significant effect on the LAI, and N placement had a significant effect on the Pn in 2017 (*P* < 0.01). However, both N source and placement did not affect LAI and Pn significantly (*P* > 0.05) in 2016. The highest LAI and Pn at the VT stage were observed in CRU application at D15 placement in both years. N source also had a significant effect on the Tr in 2016, and placement had a significant effect on the Gs in 2016. In 2016, CRU application at D15 placement significantly (*P* < 0.05) increased the Gs and Tr, whereas deep placement of urea only increased the Gs. The differences among different N fertilizer treatments were non-significant (*P* > 0.05) for SPAD in 2016, and for Gs, Tr, and SPAD in 2017.

**Table 5:** Effects of N source and placement on leaf area index (LAI), photosynthesis rate (Pn, μmol CO_2_ m^−2^ s^−1^), stomatal conductance (Gs, mmol H_2_O m^−2^ s^−1^), transpiration rate (Tr, mmol H_2_O m^−2^ s^−1^), and chlorophyll content (SPAD, %) of sweet corn at the VT stage in 2016 and 2017.

Year	Placement	N source	LAI	Pn	Gs	Tr	SPAD
			1.48 b	26.7 c	0.29 c	6.9 b	33.7 b
2016	D0	Urea	1.76 ab	42.5 a	0.40 c	7.8 b	45.2 a
		CRU	1.90 a	49.0 ab	0.38 c	10.2 ab	44.9 a
	D5	Urea	1.80 ab	46.0 b	0.38 c	7.1 b	46.9 a
		CRU	1.74 ab	52.8 ab	0.58 b	10.3 ab	43.4 a
	D15	Urea	1.73 ab	55.1 ab	0.79 a	9.2 ab	46.8 a
		CRU	1.91 a	59.5 a	0.74 a	12.3 a	48.2 a
	ANOVA						
	N source		Ns	Ns	Ns	**	Ns
	Placement		Ns	Ns	**	Ns	Ns
	N source × placement		Ns	Ns	*	Ns	Ns
2017		N0	2.06 d	37.9 b	0.32 a	3.7 a	52.4 a
	D0	Urea	2.47 bc	39.8 b	0.38 a	3.8 a	55.3 a
		CRU	2.34 c	49.4 a	0.32 a	4.1 a	54.8 a
	D5	Urea	2.56 ab	38.3 b	0.39 a	4.2 a	55.5 a
		CRU	2.50 abc	49.5 a	0.30 a	4.2 a	56.5 a
	D15	Urea	2.56 ab	42.3 b	0.34 a	4.1 a	57.2 a
		CRU	2.70 a	50.4 a	0.31 a	4.1 a	57.3 a
	ANOVA						
	N source		Ns	**	Ns	Ns	Ns
	Placement		**	Ns	Ns	Ns	Ns
	N source × placement		Ns	Ns	Ns	Ns	Ns

Data are presented as the means of three replicates. Within a column for each season, values followed by different letters indicate significant difference according to Duncan’s test at *P* = 0.05. **P < 0.01; *P < 0.05; Ns, *P* > 0.05.

### Soil inorganic N concentration

The NO_3_^−^-N and NH_4_^+^-N concentrations at the soil depths of 0–20 cm and 20–40 cm during the two years are listed in Table 6. In 2016, the effect of N source and placement on soil NH_4_^+^-N concentrations at 0–20 cm and 20–40 cm soil depths was non-significant (*P* > 0.05) at all the three growth stages. N source significantly affected the NO_3_^−^-N concentration of the soil depths of 0–20 cm at the VT stage (*P* < 0.01) and 20–40 cm at the R3 stage in 2016 (*P* < 0.05). Fertilizer placement only significantly affected the NO_3_^−^-N concentration of 0–20 cm soil depth at the VT stage in 2016 (*P* < 0.01). In 2017, N source showed significant effects on the NO_3_^−^-N concentrations of the soil depths of 0–20 cm and 20–40 cm at the three stages (*P* < 0.05) and the NH_4_^+^-N concentration of the soil depth of 0–20 cm at the VT stage and 20–40 cm at both V6 and VT stages (*P* < 0.05). The N placement also significantly affected the NO_3_^−^-N concentrations of the soil depth of 20–40 cm at the V6 and VT stages in 2017 (*P* < 0.05). The NH_4_^+^-N and NO_3_^−^-N concentrations of the soil depth of 0–20 cm in CRU application at D5 and D15 placements were significantly higher than those in urea application treatments from V6 to R3 stages in 2017 (*P* < 0.05). The NO_3_^−^-N concentrations at soil depth of 20–40 cm in CRU application at D5 and D15 placements were also significantly higher than those in urea application treatments at the VT and R3 stages in 2017 (*P* < 0.05).

**Table 6:** Effect of N source and placement on soil NH_4_^+^-N (mg kg^−1^) and NO_3_^−^-N (mg kg^−1^) concentrations in the 0–20 cm and 20–40 cm depths during main sweet corn growth periods in 2016 and 2017.

Year	Placement	N source	V6		VT		R3	
			0–20 cm	20–40 cm	0–20 cm	20–40 cm	0–20 cm	20–40 cm
		**NH**_**4**_^**+**^**-N (mg kg**^**−1**^**)**						
2016		N0	2.9 a	2.5 a	3.2 a	3.8 a	3.4 a	1.5 a
	D0	Urea	2.2 a	3.0 a	2.9 a	4.3 a	3.1 a	1.3 a
		CRU	2.3 a	3.2 a	3.4 a	3.5 a	2.5 a	1.6 a
	D5	Urea	3.0 a	3.4 a	2.9 a	3.8 a	3.2 a	1.1 a
		CRU	2.5 a	3.3 a	3.0 a	3.4 a	3.4 a	1.3 a
	D15	Urea	2.4 a	4.0 a	3.2 a	4.0 a	3.2 a	1.5 a
		CRU	3.8 a	3.5 a	3.0 a	3.6 a	2.3 a	1.5 a
	ANOVA							
	N source		Ns	Ns	Ns	Ns	Ns	Ns
	Placement		Ns	Ns	Ns	Ns	Ns	Ns
	N source × placement		Ns	Ns	Ns	Ns	Ns	Ns
		**NO**_**3**_^**−**^**-N (mg kg**^**−1**^**)**						
		N0	21.6 b	3.8 b	18.8 d	6.7 c	6.9 d	3.9 b
	D0	Urea	160.6 a	11.2 a	107.2 b	9.5 bc	84.3 bc	5.7 b
		CRU	146.8 a	11.5 a	43.1 cd	7.8 c	115.3 abc	10.5 ab
	D5	Urea	205.4 a	11.6 a	111.3 b	17.4 a	125.0 ab	13.1 a
		CRU	204.5 a	10.3 a	86.6 bc	7.1 c	78.6 c	14.2 a
	D15	Urea	184.8 a	15.0 a	185.0 a	12.3 abc	102.2 abc	9.6 ab
		CRU	190.1 a	9.8 ab	129.0 b	16.3 ab	137.5 a	16.7 a
	ANOVA							
	N source		Ns	Ns	**	Ns	Ns	*
	Placement		Ns	Ns	**	Ns	Ns	Ns
	N source × placement		Ns	Ns	Ns	*	*	Ns
		**NH**_**4**_^**+**^**-N (mg kg**^**−1**^**)**						
2017		N0	14.7 c	13.4 b	22.7 bc	4.4 c	7.4 ab	6.4 a
	D0	Urea	232.2 a	44.9 a	43.0 ab	10.9 a	10.8 ab	7.9 a
		CRU	56.4 bc	11.7 b	32.7 abc	6.7 bc	17.0 a	7.3 a
	D5	Urea	48.3 bc	13.2 b	22.9 bc	8.3 ab	5.5 b	5.5 a
		CRU	134.1 ab	11.8 b	53.6 a	5.5 bc	11.1 ab	6.8 a
	D15	Urea	30.2 bc	28.2 ab	15.2 c	8.2 ab	5.3 b	6.3 a
		CRU	77.8 bc	11.8 b	49.7 ab	5.7 bc	12.0 ab	7.9 a
	ANOVA							
	N source		Ns	**	*	**	Ns	Ns
	Placement		Ns	Ns	Ns	Ns	Ns	Ns
	N source × placement		**	Ns	Ns	Ns	Ns	Ns
		**NO**_**3**_^**–**^**N (mg kg**^**−1**^**)**						
		N0	18.3 b	7.3 b	16.2 c	4.3 d	11.3 c	12.0 c
	D0	Urea	63.9 a	38.1 a	57.1 b	7.0 cd	12.9 c	8.1 c
		CRU	27.0 b	11.2 b	58.4 b	28.7 b	38.8 ab	24.1 b
	D5	Urea	55.7 a	12.8 b	23.6 c	5.0 d	7.6 c	5.1 c
		CRU	40.6 ab	10.5 b	100.6 a	20.2 b	28.2 b	22.6 b
	D15	Urea	40.2 ab	16.7 b	34.0 bc	10.8 cd	7.4 c	4.9 c
		CRU	44.9 ab	17.3 b	83.4 a	47.0 a	47.8 a	33.3 a
	ANOVA							
	N source		*	*	**	**	**	**
	Placement		Ns	*	Ns	*	Ns	Ns
	N source × placement		Ns	*	**	Ns	Ns	Ns

Data are presented as the means of three replicates. Within a column for each season, values followed by different letters indicate significant difference according to Duncan’s test at *P* = 0.05. V6: sixth leaf stage; VT: tasseling stage; R3: harvest (milk maturity) stage. **P < 0.01; *P < 0.05; Ns, *P* > 0.05.

## Discussion

Considerable studies have been carried out to compare the effects of varying fertilizer application methods on nutrient use efficiency and crop productivity. Generally, deep fertilizer placement in soil can increase fertilizer use efficiency and crop yield compared with surface or shallow application^14,31,32,35–37^. For instance, Yao *et al*.^31^ indicated that deep N placement in paddy fields increased the rice yield and NUE by 10% and 55%, respectively, compared with surface broadcasting. Guo *et al*.^37^ showed that deep CRU placement increases the N uptake and NUE of maize on clay loam soil. In the present study, deep CRU placement significantly improved the NUE and fresh ear yield of sweet corn by 109.04% and 9.43%, respectively compared with CRU application at 0 cm depth, averaged across two years (Table 1). These results are similar to those of previous studies on maize, soybean and rice^32,34,37^. Whilst, the effect of different N placements was non-significant on NUE and fresh ear yield of sweet corn when conventional urea was used as N source (Table 1) and these findings contradict with the results reported by Smith *et al*.^35^. Yao *et al*.^31^ also reported that deep urea placement in intensive rice cropping system can provide NH_4_^+^-N concentration in the soil during the early growth stage and extend N availability duration for two months. The different results for urea in the present study might be due to variations in environmental conditions such as rainfall pattern and soil properties, cultivars, and agronomic practices (e.g. fertilization, irrigation, and tillage). Moreover, greater NUE and fresh ear yield with deep placement of CRU might be attributed to enhancement in root morphology attributes in the present study. Compared with D0, CRU application at D15 significantly increased the root surface area and root volume by 48.19% and 73.90%, respectively averaged across two years. Nevertheless, these two root attributes remained statistically similar for urea application at D0 and D15. These better performance of deep placement of CRU over urea has previously been reported in rice^36^, possibly due to slow release of CRU^22^.

The results of the present study revealed significant variations in soil NO_3_^−^-N and NH_4_^+^-N concentrations (Table 6). thereby indicating that environment factors, such as temperature and precipitation, regulated soil inorganic N dynamics in sweet corn field. The soil NH_4_^+^-N concentrations at the three stages in 2016 ranged from 2.2 mg kg^−1^ to 3.8 mg kg^−1^ at the soil depth of 0–20 cm and from 1.1 mg kg^−1^ to 4.0 mg kg^−1^ at the soil depth of 20–40 cm. These values were significantly lower than those in 2017 (5.3–232.2 mg kg^−1^ at 0–20 cm soil depth and 4.4–44.9 mg kg^−1^ at 20–40 cm soil depth; Table 6). However, the soil NO_3_^−^-N concentrations at the three stages in 2016, especially at the soil depth of 0–20 cm, were higher than those in 2017. Such results can be ascribed to the differences in climatic conditions, especially precipitation, and soil properties between two fields. In the present study, an increase in the soil inorganic N concentration (especially NO_3_^−^-N content in 0–20 cm soil layers) was observed at the later growth stage of sweet corn under CRU deep placement (D15) compared with that under D0 or D5 (Table 6). This increase was possibly due to the increase in the contact of N fertilizer granules with soil and decrease in N losses through runoff and NH_3_ volatilization^30,31,38^. Therefore, the enrichment of plant available N concentration also promoted the aboveground dry matter accumulation and N uptake in sweet corn plants (Tables 2 and 3) and ultimately improved the NUE and fresh ear yield (Table 1 and S1). Better root growth (Table 4 and S2) and leaf physiological functions (LAI, Pn, Gs, Tr, and SPAD) (Table 5 and S2) at the VT stage may also explain the higher NUE, total N uptake, and fresh ear yield under deep placement of CRU.

In our study, deep placement of CRU and urea significantly affected the soil inorganic N concentration during the different growth stages of sweet corn, which can be associated with the nitrification–denitrification processes in soil^39^. However, the magnitude of nitrification–denitrification under deep CRU and urea placement is unclear. Therefore, the responses of N processes to deep CRU and urea placement need to be explored. In general, the available N in the plant root zone (0–40 cm soil layer) soil samples and NO_3_^−^-N concentration of CRU treatment were higher than those in urea treatments, especially for D15 placement (Table 6). A possible reason for such results might be the losses of urea-N through leaching into underground water. Compared with urea, the release of N in CRU is slow, and only a small amount of N is leached into underground water^25^. This also indicates that CRU can increase the amount of available N in the plant root zone and decrease the risk of N losses.

## Conclusions

The results of this study revealed that deep placement of urea did not significantly affect the fresh ear yield and NUE of sweet corn. However, deep placement of CRU at 15 cm depth significantly enhanced the fresh sweet corn ear yield and NUE. The higher fresh ear yield and NUE of sweet corn under deep CRU placement were attributed to improved root growth, better leaf physiological functions and increased soil NO_3_^−^-N and NH_4_^+^-N concentrations, especially during the later growth stages of sweet corn.

## Materials and Methods

### Experimental site

**Figure 2:**
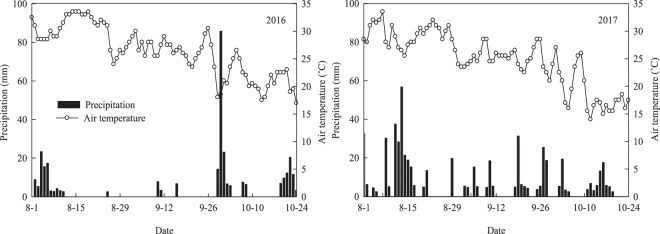
Precipitation and average daily air temperature during sweet corn growing seasons in 2016 and 2017.

The experiments were carried out in two different fields (1.8 km apart) located in Ezhou City (30°21′ N, 114°45′ E), Hubei Province, central China during the sweet corn growing seasons (August–October) in 2016 and 2017. The soil type in the two fields was typical fluvo-aquic soil (entisol according to USDA classification^40^). The soil physico-chemical properties of the soil layers of 0–20 cm and 20–40 cm prior to experiments are presented in Table 7. The atmospheric temperature and rainfall during two years of sweet corn growing seasons are provided in Fig. 2. Compared with the average annual climatic conditions in the region, the total rainfall during the sweet corn growth season in 2016 (272 mm) was less than that in 2017 (530 mm). Minimal rainfall was observed from mid-August to late September in 2016.

**Table 7:** Physical and chemical properties of soils (0–20 cm and 20–40 cm depths) prior to sowing in 2016 and 2017.

Year	Depth (cm)	Organic matter (g kg^−1^)	Total N (g kg^−1^)	NH_4_^+^-N (mg kg^−1^)	NO_3_^—^N (mg kg^−1^)	Available P (mg kg^−1^)	Available K (mg kg^−1^)	pH
2016	0–20	15.1	1.3	2.7	15.1	14.7	197.9	7.8
	20–40	10.3	0.7	2.3	7.6	2.6	103.4	7.9
2017	0–20	28.5	2.6	9.3	10.3	26.4	182.5	7.3
	20–40	20.1	1.4	7.8	4.1	16.9	114.0	7.2

### Experimental design and management

Experiment was comprised of seven treatments, including two N sources (CRU and conventional urea), three fertilization placement depths (0 cm as D0, 5 cm as D5, and 15 cm as D15), and a control (N0) without N fertilization treatment. The treatments were arranged in a randomized block design with three replications. The planting pattern of sweet corn was wide-narrow row planting, which was consistent with the conventional growing conditions in China. Urea and CRU were applied in narrow rills of the desired depth (0, 5, and 15 cm) ditched manually between two maize rows sown at 40 cm distance. The soil was leveled after fertilizer placement. CRU is a polymer-coated fertilizer that is manufactured and supplied by the South China Agricultural University and contains 44% N with the nutrient release period of 60 days, while conventional urea containing 46% N was provided by the Hubei Sanning Chemical Co., Ltd. (Hubei, China). The application rates of nutrients in each treatment except N0 included 180 kg N ha^−1^, 45 kg P_2_O_5_ ha^−1^, and 90 kg K_2_O ha^−1^, which were similar to the local farming recommendations. The fertilizers were all applied at the basal dose 1 day before sowing. Each plot area for sweet corn plantation was 20 m^2^ (length × width of 8.3 m × 2.4 m). Two sweet corn varieties, namely, Fotian 2 (in 2016) and Jintian 678 (in 2017), which were selected by a farmer according to the market, were used in the study. The planting density was 50,000 plants ha^−1^ (narrow row spacing of 40 cm, wide row spacing of 80 cm, and plant spacing of 33 cm) in both years. The sweet corn seeds were sown on August 1, 2016 and August 3, 2017, and the fresh ears were harvested on October 18, 2016 and October 19, 2017. In addition to the treatments under the study, all the other field management practices, including variety selection and weed, disease, and insect control, throughout the growing season were similar and followed the recommended practices by the local agriculture department.

### Sampling and analysis

At the sixth leaf (V6; 25 days after sowing [DAS] in 2016, 26 DAS in 2017), tasseling (VT; 50 DAS in 2016, 55 DAS in 2017), and harvest (milk maturity) stages (R3; 75 DAS in 2016, 79 DAS in 2017), eight soil subsamples near the plant row (four on each side) were collected in each plot at 0–20 cm and 20–40 cm depths using a stainless steel auger and then mixed to obtain a single composite sample of the same layer. The soil samples were extracted with 2 M KCl (1:10 soil to extractant ratio, 30 min of shaking), and NO_3_^−^-N and NH_4_^+^-N concentrations were measured using an automated flow injection analyzer (AA3, Bran and Luebbe, Norderstedt, Germany).

At the same three stages, the aboveground tissue samples were collected from five representative plants in each replicate of the treatment. The leaves, stems and leaf sheaths, tassels, bracts, rachis, and grains were separated and oven dried for 30 min at 105 °C and at 70 °C to a constant weight in determining the dry matter weight. The N concentration of each part was analyzed using H_2_SO_4_–H_2_O_2_ digestion and a continuous flow injection analyzer (AA3, Bran and Luebbe, Norderstedt, Germany). N uptake was calculated by multiplying the dry matter weight with their respective concentrations in different aboveground parts.

Similarly, at the V6 and VT stage, the three representative roots sampled from each plot were carefully washed and used to measure the root morphology, including root L, surface area, and volume, via WinRhizo software (WinRhizo, Regent Ltd., Canada).

Leaf length and width were measured at the VT stage as described above from the five representative plants, and the leaf area (LA) was then calculated as follows: LA = length × width × *K*, where *K* is 0.75 for fully expanded leaves and 0.5 for incompletely expanded leaves^41,42^. The leaf area index (LAI) was calculated as the sum of the green LA of the five plants divided by the area occupied by those plants. Five sweet corn ear leaves at the VT stage were selected in each plot in measuring the Pn, Gs, and Tr by using a Li-6400XT portable photosynthesis system (Li-Cor, USA) in the morning at 9:00–11:00. The chlorophyll content (SPAD) of the ear leaves was verified with a SPAD-502 chlorophyll meter (Minolta, Osaka, Japan). At the R3 stage, 40 consecutive ears were harvested in each plot to determine the fresh ear yield.

Nitrogen release rates of CRU were tested in a laboratory by incubating in 25 °C water^43^. In brief, 10.0 g of CRU sample were placed in a glass bottle containing 200 ml of distilled water with three replicates, and then kept in a constant temperature incubator at 25 °C. The solution samples were collected at 1, 3, 5, 7, 10, 13, 16, 19, 22, 28, 35, 42, 49, 56, and 63 days or when the cumulative N release rate was more than 80%. The released N content from the CRU was determined using the Kjeldahl method, followed by calculating rates of N release from the CRU.

### Calculations

The NUE was calculated according to López-Bellido *et al*.^44^:

$${\rm{NUE}}( \% )=({T}_{N}\mbox{--}{T}_{0})/{F}_{N}\times 100 \% ,$$

where *T*_*N*_ and *T*_0_ are the total N uptake at milk harvest stage from the N-fertilized and unfertilized N0 plots (kg N ha^−1^), respectively, and *F*_*N*_ is the N fertilization rate (kg N ha^−1^).

### Data analysis

The experimental data were statistically analyzed using SPSS 16.0 software. Two-way ANOVA was used to evaluate the effects of N source, placement, and interactions among factors. The differences among treatments were calculated using the Duncan method at *P* = 0.05. The figures were prepared via SigmaPlot 10.0 software.
